# The 2 + 1 paradigm: an efficient algorithm for central reading of Mayo endoscopic subscores in global multicenter phase 3 ulcerative colitis clinical trials

**DOI:** 10.1093/gastro/gov024

**Published:** 2015-09-11

**Authors:** Harris A. Ahmad, Klaus Gottlieb, Fez Hussain

**Affiliations:** ^1^Rheumatology and Gastroenterology, BioClinica Inc., Princeton, NJ, USA and; ^2^Immunology and Internal Medicine, Quintiles Inc., Research Triangle Park, NC, USA

**Keywords:** ulcerative colitis, endoscopy, clinical trials

## Abstract

Despite its importance and potential impact in clinical trials, central reading continues to be an under-represented topic in the literature about inflammatory bowel disease (IBD) clinical trials. Although several IBD studies have incorporated central reading to date, none have fully detailed the specific methodology with which the reads were conducted. Here we outline key principles for designing an efficient central reading paradigm for an ulcerative colitis (UC) study that addresses regulatory, operational and clinical expectations. As a step towards standardization of read methodology for the growing number of multicenter phase 3 clinical trials in IBD, we have applied these principles to the design of an optimal read methodology that we call the ‘2 + 1 paradigm.’ The 2 + 1 paradigm involves the use of both site and central readers, validated scoring criteria and multiple measures for blinding readers, all of which contribute to reducing bias and generating a reliable endoscopic subscore that reflects endoscopic disease severity. The paradigm can be utilized while maintaining a practical workflow compatible with an operationally feasible clinical trial. The 2 + 1 paradigm represents a logical approach to endoscopic assessment in IBD clinical trials, one that should be considered attractive to prospective sponsors, contract research organizations, key opinion leaders and regulatory authorities and be ready for implementation and further evaluation.

## Introduction

Central reading continues to be an under-represented topic in the literature on inflammatory bowel disease (IBD) clinical trials compared with trials for other disease areas such as oncology, osteoporosis and rheumatology [1–6]. Central reading of videos captured at sigmoidscopy or colonoscopy have been demonstrated to have an impact on the measurable differences in disease activity observed between placebo and treatment groups [7,8]. For example, in a recent clinical trial designed to evaluate treatment with mesalamine, Feagan *et al*.demonstrated a larger separation between placebo and active drug with the post-hoc use of central reading [7]. Surprisingly, Kobayashi *et al.* reported a narrowing of this separation instead, which illustrated that central reading per se does not necessarily translate into a reduction of noise [8]. The reasons for these divergent results are likely multifactorial. In contrast to Feagan *et al.*,who used a single central reader, Kobayashi *et al*. incorporated a consensus mechanism into their read approach while utilizing endoscopic still images rather than video endoscopy.

While the manner in which central reads are conducted is quite variable among IBD trials and trials in other therapeutic areas, the call for utilizing central reading in clinical trials is clearly stated in the FDA’s March 2015 revised draft guidance *Clinical Trial Imaging Endpoint Process Standards* [9]: *‘*As compared to site-based image interpretations in multicenter clinical trials, we anticipate that a centralized image interpretation process may provide more verifiable and uniform reader training as well as ongoing management of reader performance, helping to ensure quality control of the images and their interpretation and to decrease variability in image interpretations, leading to a more precise estimate of treatment effect*.’*

However, in the same guidance, the FDA also stresses the value of a site reader by stating: *‘*Nevertheless, the overarching trial design features and the other previously described features may justify the use of site-based imaging interpretations, even in large phase 3 multicenter clinical trials, so long as blinding of image interpretation to treatment can be assured or bias is otherwise controlled*.’*

Although a handful of IBD studies have incorporated central reading to date, none have fully detailed the specific methodology with which the reads were conducted [7,8,10,11]. Ahmad *et al*. recently reported on a variety of distinct read approaches for IBD clinical trials, each of which varied in the stepwise determination of a final endoscopic score [12]. These central reading approaches involve the use of one or more central readers and, in some instances, a site reader. It is unlikely that a single approach to read design will be suitable for all clinical trials given the large number of trial variables (e.g. endpoints under assessment, need for regulatory submission, scope of the trial design and number of study sites). Nonetheless, while tactical approaches may vary between studies, certain key principles can be applied to all central reading paradigms designed for IBD clinical trials.

As a step toward standardizing read methodology for the growing number of multicenter phase 3 clinical trials in IBD, we present a reading approach for ulcerative colitis (UC) studies involving both central and site readers that we call the ‘2 + 1 paradigm.’ This paradigm is based on the theoretical foundations of read paradigms rooted in voting algorithms [13] and practical clinical trial considerations.

## Five key principles for designing an efficient read paradigm

There are five principles to consider when choosing an efficient central read paradigm for a UC study. These principles, summarized below, address regulatory, operational and clinical expectations.

1. **Use of a clinical scoring scheme that has strong construct validity** (e.g. the Mayo Clinic Score (MCS) endoscopic subscore criteria, **Table 1** [14)).
2. **Implementation of a quality-control process to assure suitable video capture that accurately reflects disease in the affected bowel.** The quality of the source video captured at a site is critical to all downstream disease evaluations. Engagement with site endoscopists is a prerequisite for obtaining optimal video recordings. The site endoscopist must be fully involved in the read paradigm, adequately trained in the protocol expectations and preferred acquisition technique and fully understand the selected scoring system as it applies to the central and site readers as discussed in Gottlieb and Hussain [13]. Once being received at a central core lab and reviewed for quality, suboptimal recordings should be discussed in detail with the site endoscopist in order to improve video quality on follow-up endoscopies. Both the central lab and site endoscopist should be committed to accurately achieving quality depiction of the mucosa.
3. **Reducing/eliminating sources of bias in the interpretation of the video endoscopy.** Bias can be introduced by unblinded readers when evaluating patient eligibility and drug efficacy and may reduce the observable differences between drug and placebo [7,8]. To eliminate key sources of bias, the read methodology must preserve blinding with regard to the purpose of the video endoscopy based on its chronicity (i.e. the reader must not be able to distinguish whether the patient visit is for screening, establishment of a baseline measurement or a follow-up measurement). Readers should also be blinded to patient demographics, treatment arm and the score of any other participating reader (site or central). Furthermore, in the setting of a discordant subject score, a central reader should not be notified about his or her read designation in the paradigm (i.e. first reader, second reader or adjudicator).
4. **Implementation of video endoscopy review process that generates a quantifiable reflection of disease activity with reasonable accuracy and reproducibility.** Variability in scoring between site endoscopists and central readers can be significant and may alter the outcomes of a study [7,8]. Therefore, arriving at an accurate reflection of disease activity with a chosen scoring method should involve both parties with a reasonably time-efficient method for determining a final score that most represents the endoscopic severity.
5. **Operational feasibility that facilitates proper and uninterrupted clinical trial conduct.** While screening periods are typically two to three weeks in length, completion and reading of video endoscopy are usually completed as the last study examination. This leaves only several days for video acquisition and score reporting. This time constraint necessitates a paradigm of reduced complexity that accommodates multiple central readers and circumvents operational roadblocks.

**Table 1.** Mayo Clinic Score endoscopic subscore criteria

Score	Definition
0	Normal or inactive disease
1	Mild disease (erythema, decreased vascular pattern, mild friability)
2	Moderate disease (marked erythema, lack of vascular pattern, friability, erosions)
3	Severe disease (spontaneous bleeding, ulceration)

## The 2 + 1 paradigm: an efficient algorithm for phase 3 Trials

**Figure 1.**
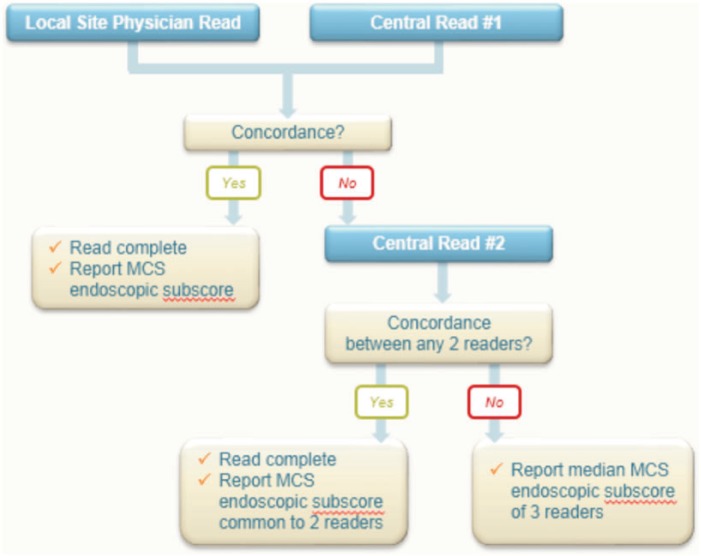
The 2 + 1 paradigm: an efficient algorithm for phase 3 ulcerative colitis trials

The following central reading approach, which we call the ‘2 + 1 paradigm’ (summarized in **Figure 1**), has been established for several large phase 3 UC clinical trials. The approach involves the use of two independent readers (one site endoscopist and one central gastroenterologist), followed by one central reader providing a score in the case of score disagreement. Using this approach, the site score is submitted to a central imaging core lab along with the accompanying digitally recorded and electronically submitted video endoscopy. Once the video endoscopy has been transmitted by the site, it is electronically allocated for reading by a qualified, experienced gastroenterologist. The central imaging core lab typically draws from a pool of similarly trained gastroenterologists with similar backgrounds who will apply the same scoring criteria as the site endoscopist.

If there is agreement between the site reader and the blinded central reader (central read #1) for the overall MCS endoscopic subscore, a final MCS endoscopic subscore is reported. If there is discordance between the site reader and the central reader for the overall MCS endoscopy subscore, the video endoscopy will be assigned to a second blinded reader (central read #2) chosen from a pool of remaining central readers who are blinded to the scores of the site reader and the first central reader. The second central reader is also blinded to the number of times that the video endoscopy has been evaluated and will score it according to the same criteria applied by the site and previous central reader. From a total of the three overall endoscopic MCS subscores, the score with which two readers agree will be reported as the final overall MCS endoscopic subscore.

In the unlikely event that no two readers agree on a final overall MCS endoscopic subscore, the median score of all three completed reads (i.e. site read, central read #1 and central read #2) will be chosen as the final reported overall MCS endoscopic subscore. Scenarios of final score determinations in such a setting are summarized in **Table 2**.

**Table 2.** Final score determination through selection of the median score between three readers

Site endoscopist	Central reader 1	Central reader 2	Final score
3	2	1	2
3	2	0	2
3	1	0	1
2	1	0	1
2	1	3	2
2	3	1	2

In the event that a submitted video endoscopy is determined to be ‘not readable’ by the second central reader, the reported overall MCS endoscopic subscore will default to that of the site reader. Given the burden of an endoscopy procedure coupled with the operational urgency for determining clinical trial eligibility, this method is the most favorable at the current time.

## Discussion

The 2 + 1 paradigm utilizes the five key principles outlined in this paper to deliver a sound, centrally based imaging and reading methodology for UC trials. As many previous studies have done, one might suggest exclusion of the site endoscopist entirely from disease evaluation. However, there is a fundamental difference between endoscopist-generated video endoscopy and technologist-acquired imaging such as radiographs, magnetic resonance imaging (MRI) or computed tomography (CT). For example, non-IBD clinical trials utilizing MRI or CT imaging are usually dependent upon the technologist’s skill to generate quality images and may even rely on an automated assessment of computer-generated images to arrive at a quantifiable score of disease activity. In contrast, IBD trials utilizing video endoscopy to assess disease activity depend on the motivation, attention and skill of the site endoscopist to drive the imaging instrument, decide on the area and extent of focus and generate the quality and quantity of video content necessary for accurate disease evaluation by a central reader.

Furthermore, site endoscopists who are expected to submit their endoscopic score of disease severity may experience a personal motivation to generate videos of sufficient quality to assure that a central reader will corroborate their own interpretation (Hawthorne effect) [15]. It is important to note that tasking the site endoscopist with scoring requires adequate pre-study training similar to that of the central reader, which could be accomplished via investigator meetings, recorded video training sessions and scoring exercises prior to the start of a trial. Consequently, by assigning the site reader as a critical resource, the 2 + 1 read paradigm promotes high-quality endoscopic video capture and enhances accuracy in the assessment of disease activity.

Another benefit of the 2 + 1 paradigm is the elimination of common biases—of which regulatory agencies are keenly aware—particularly central reader bias associated with knowledge of the protocol-defined timing of the endoscopic video, patient and site-specific identifiers as well as knowledge of another reader’s interpretation. Both the site and central reader independently assess a single video endoscopy using the MCS endoscopic subscore, with the central reader being blinded to treatment assignment, patient identifiers and chronological nature of the endoscopy.

Utilization of a blinded second central reader (only in the event of score discordance between the site and first central reader) eliminates bias associated with the need for an adjudicator who is often required to choose a score between that provided by the site and central readers. By blinding all central readers to patient and video sequence identifiers, the second central reader will also not be biased toward a certain score, especially when relying on a scale of only four scores (0, 1, 2 or 3). The unlikely introduction of a distinct third score is efficiently and conservatively resolved by deferring to the median score, which minimizes study delays associated with reconciliation of discrepant endoscopic scores from multiple readers yet allows an unbiased second central reader assessment.

Finally, with evidence from multiple studies that reproducibility for the Mayo endoscopic subscore is suboptimal [16,17], a single central reader may introduce significant error and bias into a clinical trial. In contrast, the 2 + 1 paradigm seeks to arrive at the most reliable endoscopic subscore in a practical manner by leveraging the accuracy and scientific expertise of more than one reader while limiting reader bias. Eliminating these common sources of reader bias is expected to diminish the risks of improper study enrollment and variability in treatment response [7,8].

## Conclusion

The 2 + 1 paradigm for phase 3 UC trials described here adheres to the five key principles of an effective clinical trial read paradigm in IBD and is consistent with regulatory guidance calling for central reading with a reasonable rationale for utilizing site reading [9]. While utilizing validated scoring criteria, the paradigm blinds the central reader to the chronicity of time points, engages the site reader in the assessment of the patient, blinds all readers to other reader scores and their specific role and produces an endoscopic subscore that is reflective of endoscopic disease severity. The paradigm can be accomplished while maintaining a practical workflow compatible with an operationally feasible clinical trial. With multiple measures aimed at reducing bias, we believe the 2 + 1 paradigm to be a logical approach to endoscopic assessment in IBD clinical trials, one that should be considered attractive to prospective sponsors, contract research organizations, key opinion leaders and regulatory authorities and be ready for implementation and further evaluation.
